# Between the clinic and the community: a qualitative study of logics of action on social determinants of health in general practices serving disadvantaged communities

**DOI:** 10.1186/s12889-026-27790-7

**Published:** 2026-05-28

**Authors:** Mihirini Sirisena, Alisha Gupta, Sam Redgate, Jennie Sofia Portice, Sameena Hassan, Sarah Sowden

**Affiliations:** 1 Population Health Sciences Institute, Newcastle University, Newcastle, UK; 2 Applied Research Collaboration (ARC) North East and North Cumbria (NENC), National Institute for Health and Care Research (NIHR), Newcastle, UK; 3 School of Communities and Education, Northumbria University, Newcastle, UK; 4 Deep End Network North East North Cumbria, Gateshead, UK

**Keywords:** General practice, Social determinants of health, Socio-economic factors, Qualitative research, Deep End, United Kingdom

## Abstract

**Background:**

Persistent socioeconomic inequalities in health remain a major public health challenge. Although the social determinants of health (SDOH) are widely recognised as foundational drivers of health inequities, translating structural understandings into sustained system-level action remains difficult. Primary care is increasingly positioned as a key site for advancing health equity; however, practitioners’ orientations toward SDOH vary and are shaped by institutional, organisational, and ideological contexts. This study examines how general practices operationalise action on SDOH when provided with dedicated resources and flexibility to respond within socioeconomically disadvantaged settings.

**Methods:**

This qualitative study explored the implementation of CareDEEP, a 12-month initiative within the Deep End Network of North East North Cumbria (England), which provided funding and peer support to general practices serving highly deprived populations. Ten practices designed and implemented locally determined initiatives addressing SDOH. Drawing on realist-informed qualitative analysis, we examined how contextual configurations shaped practice responses. Data included monitoring reports, observational data from peer meetings, and interviews with participating staff. Analysis was informed by theoretical frameworks on SDOH discourses (Raphael) and functional, analytical, and structural approaches to action (Brassolotto et al.).

**Results:**

Practices demonstrated distinct but patterned “logics of action” in how SDOH were interpreted and addressed. These ranged from functionally oriented service-level responses to more analytically or structurally framed initiatives. The orientation adopted was shaped by interacting contextual factors, including workforce capacity, leadership, prior exposure to health inequality work, organisational pressures, and local partnership infrastructure.

**Conclusions:**

General practice can function as a site of public health implementation for action on SDOH, but responses are mediated by contextual, including ideological, factors. Interventions seeking to support structural engagement in primary care must attend not only to resources but also to the organisational and discursive conditions shaping practitioner reasoning. Understanding these logics of action is critical for designing policies that strengthen primary care’s contribution to health equity.

**Supplementary Information:**

The online version contains supplementary material available at 10.1186/s12889-026-27790-7.

## Introduction

Persistent socioeconomic inequalities in health remain a central challenge for public health systems [20]. While the social determinants of health (SDOH) are widely recognised as foundational drivers of population health [25, 32], translating structural understandings of inequality into sustained system-level action remains difficult [2, 35]. Recent calls for whole-system approaches emphasise the need for coordinated action across sectors and governance levels [12, 15, 33]. However, policy and practice often default to individualised or service-level interventions, reflecting persistent ideological and organisational constraints [23, 31, 35].

Among sites of action on adverse SDOH, primary care is positioned as a key site for advancing health equity, where its population reach, continuity, and relational capacity have been highlighted as bearing the potential to bridge clinical services and community contexts [33]. However, research suggests that practitioners’ understandings of health inequalities vary considerably and these understandings influence whether action on inequality is framed as mitigation, advocacy, or structural reform [1, 10]. Considering these diverse conceptualisations of SDOH, Raphael [30] identifies seven discourses that frame action: identifying individuals in need of health services,focusing on modifiable medical or behavioural risk factors,emphasising material living conditions; recognising variation by group membership (e.g., class, gender, race); linking social determinants to public policy decisions; analysing the impact of economic and political structures; and interrogating the power and influence of groups benefiting from inequalities [30]. Brassolotto et al. [4] elucidates that actions underlying these discourses generally follow one of three approaches. Functional approaches focus on service delivery and individual behaviour change, framing social determinants as modifiable risk factors [4]. Analytical approaches highlight the impact of living conditions on health and direct action toward reducing the association between adverse conditions and ill health. Structural approaches conceptualise social determinants as markers of entrenched systemic inequities and seek to initiate structural change. These frameworks provide a lens to examine the logics of action through which health practitioners interpret and respond to SDOH.

In practice, research notes that, often, practitioners operate within discourses that focus on individual need and modifiable medical or behavioural risk factors [22, 23, 31]. The majority of practice, which falls within the realm of functionalist approaches to addressing SDOH, is noted to adopt an individualising lens, emphasising personal responsibility over structural causes, a view reinforced by dominant biomedical discourses, organisational practices, and external pressures privileging acute care [4, 23, 29]. Yet, some practitioners lean towards structural-analytical approaches, viewing inequalities as symptomatic of broader systemic inequities [22]. McMahon [22] illustrates that such orientations are influenced by personal and professional exposure to social disadvantage, supportive leadership, cross-sector collaboration, and conducive relational dynamics within organisations. This existing evidence base indicates the importance of understanding contextual determinants when designing approaches to involve general practice in addressing SDOH. This paper uses CareDEEP – a project developed by the Deep End Network of General Practitioners in the North East and North Cumbria, as an empirical lens to examine how health system actors operationalise action on SDOH in socioeconomically disadvantaged primary care environments. By situating general practice as a site of public health implementation, the study explores how institutional pressures, practitioner reasoning, and prevailing inequality discourses interact to shape distinct orientations toward health equity.

The Deep End Network is a self-organised movement of general practices serving the most socioeconomically disadvantaged communities, aiming to strengthen advocacy, share learning, and foreground health inequalities within primary care [5, 18, 37, 38]^1^. In England, the Deep End Network of North East North Cumbria (DEN NENC) was established in 2020 to support practices serving highly deprived populations. DEN NENC encompasses 52 practices, where at least 50% of patients live in the most disadvantaged 15% of neighbourhoods, according to Indices of Multiple Deprivation (IMD) data [14, 38]^2^. Located in one of England’s most socioeconomically disadvantaged regions—where rates of ill health and premature mortality are among the highest nationally (NHS, 2026)—DEN NENC represents a targeted initiative to mitigate health inequalities by directing resources, support, and opportunities to communities in greatest need [8]. CareDEEP, an initiative developed by DEN NENC, provided flexible funding and peer support to enable practices to design responses to SDOH within their communities. In this paper, we examine the contextual configurations that shape practices’ approaches to CareDEEP to identify the underlying logics of action. We show how practitioners interpret social determinants and navigate organisational and structural constraints when developing their interventions. By connecting theoretical discourses with empirical practice, the study illuminates the interplay between ideology, organisational context, and practitioner decision-making in primary care, offering transferable insights into how interventions can better support structural approaches to health equity.

## Methods

### Study design

We conducted a theory-informed qualitative study guided by realist principles to examine how contextual configurations shape general practices’ responses to SDOH within socioeconomically disadvantaged primary care settings. Realist logic posits that interventions operate through the introduction of new resources into existing social relationships, within which certain contextual conditions activate mechanisms for change by altering participants’ capacities, constraints, and choices [7, 13, 16]. As CareDEEP gave GPs autonomy to design their actions, understanding what informed those actions was essential to making sense of the underlying reasoning mechanisms. We therefore focused on contextual conditions as a key analytical lens. We conceptualised contextual conditions both interpretively—as the conditions within which actions acquire meaning [9] – and configurationally, a web of causal processes consisting of institutional, ideological, and organisational factors which interact dynamically with reasoning underlying interventions [13]. This dual framing enabled us to show how practitioners made sense of performance regimes, workforce pressures, and socio-economic disadvantage, and how these interpretive processes were shaped by—and in turn interacted with—practitioner reasoning to produce distinct courses of action.

### Setting and intervention

The Deep End Network North East and North Cumbria (DEN NENC) comprises general practices serving communities with high levels of socioeconomic deprivation [26, 38]. The region experiences significant health inequalities relative to national averages (NHS 2026). Deep End networks have sought to foreground the experience of practices working in contexts of “blanket deprivation” and to strengthen collective voice and peer support [37, 38].

CareDEEP was developed in response to priorities identified by general practice staff through co-design research [38] and adopted a bottom-up approach to addressing social determinants of health within DEN NENC. Applying the learning from the co-design research, CareDEEP looked to create conditions in which practices could determine how social determinants were understood and operationalised in their local contexts. Practices were required to introduce an additional service or process within their setting that explicitly engaged with social determinants of health. Within this requirement, practices retained flexibility to design, adapt, and refine their initiatives in response to local needs and emerging implementation challenges. In line with the co-design and evaluative ethos of the NENC Deep End Network, participating practices were required to engage with the associated research to contribute to further learning. This included reviewing the participant information sheet, liaising with the researcher, and making an informed decision regarding participation.

CareDEEP supported ten DEN NENC practices over a 12-month period (April 2024–April 2025) to design and implement initiatives aimed at addressing social determinants affecting their patient populations. All DEN NENC practices were invited to submit expressions of interest (EOIs) on a first-come, first-served basis for the 10 funded places available in the initial wave. Eleven practices submitted EOIs; available funding was extended to support all 11. One practice subsequently withdrew due to recruitment challenges, resulting in 10 practices completing the programme. Each participating practice received approximately £38,000 in funding. Additional support was provided through practical input from the DEN NENC Steering Group and the CareDEEP project management team, as well as structured opportunities for shared learning. Administrative processes were designed to be straightforward. Practices submitted brief quarterly monitoring forms, upon receipt of which staged funding payments were released. Monitoring forms captured information on project activities and progress and were intended to support ongoing reflection. Participants also attended pre-arranged virtual peer support meetings every two months to share learning and discuss implementation challenges.

### Data collection and analysis

We generated data for the study through multiple sources, including document analysis, interviews, observations, and a focus group. Data collection continued until all participants had the opportunity to contribute and no new avenues for data generation remained.

We conducted initial exploratory interviews (*n* = 9) with general practices participating in CareDEEP and those involved in CareDEEP’s project management at DEN NENC. These were complemented by a document analysis of expressions of interest and monitoring forms, and fieldnotes from observations at peer meetings, network meetings and practices. Insights from these sources informed the topic guide, which was used in a second round of interviews with CareDEEP practices. All interviews and the focus group were conducted online, recorded, anonymised, and then transcribed by a professional service. Topic guides were used to inform the interviews and focus groups and are provided as supplementary materials. Table 1 presents an overview of data collection activities.

**Table 1:** Overview of data collection

Round	Type of data collection	Data source	Number
Round 1 (September 23- February 24)	Number of interviews (*N* = 9) Number of participants (*N* = 12)	General Practitioners	*N* = 5
		Practice Managers	*N* = 2
		Voluntary and Charity Sector Practitioners	*N* = 2
		Social Prescribing Link Worker	*N* = 1
		CareDEEP project management Team	*N* = 2
Round 2 (June 24 – April 25)	Document Analysis	Expressions of Interest	*N* = 10
		Monitoring Forms	*N* = 24
	Observations	At peer support meetings, networking events, and practice settings	Approx. 17 h
	Number of Interviews (*N* = 10) Number of participants (*N* = 14)	General Practitioners	*N* = 5
		Practice Managers	*N* = 5
		Voluntary and Charity Sector Practitioners	*N* = 1
		CareDEEP project management Team	*N* = 1
		CareDEEP workers	*N* = 2
	Focus Group	Practice Managers and Voluntary and Charity Sector Practitioners	*N* = 1 (4 participants)

We analysed the data iteratively alongside ongoing data generation, using NVivo 14 to support the process [6]. Adopting a grounded approach [36], we identified emerging themes from the initial interviews, which related to how participants conceptualised SDOH and their role in addressing them. As analysis progressed, interpretation was refined through engagement with scholarship on SDOH discourse [30, 32], ideological and organisational determinants of inequality strategies [31], and professional constructions of health inequality in primary care [1].

Rather than treating deprivation as a static background condition, we examined how institutional pressures—including workforce strain [27], resource differentials [11], and system reform [28]—interacted with practitioner reasoning to shape responses. Through iterative comparison and triangulation across interviews, observations, and documents, we identified three trajectories of action, each underpinned by a distinct “logics of action”: an interpretive framework combining normative commitments, feasibility assessments, and institutional constraints, shaping how action on SDOH is enacted in practice.

Ethical approval was obtained from Newcastle University Research Ethics Committee (Ref: 46,731/2023). All participants provided informed consent prior to participation and recording.

## Results

The core premise underlying CareDEEP is a call to action for DEN NENC general practices to address the social determinants affecting their patients’ health and wellbeing. This call is a departure from general practitioners’ everyday tasks surrounding biomedical needs of their patients. Significant socio-economic disadvantage, which constitute the background on which the Deep End practices operate in, is brought to the foreground through CareDEEP, which leads to the emergence of a novel situation where the Deep End general practices are invited to carve out a new role. In translating this call to action, our findings indicate variability in the pathways pursued by CareDEEP practices. Table 2 provides a summary of the initiatives undertaken by the practices.

**Table 2:** Summary of CareDEEP initiatives developed by DEN NENC general practices

Practice	Practice characteristics	Initiatives
A	Urban, coastal practice serving approx. 18,000 patients^*^	Care-Co-ordinator to educate patients to improve screening attendance (AAA, breast, cervical, and bowel cancer), lung health checks
B	Urban, inland practice serving approx. 14,000 patients	Specialist social prescribing for patients with persistent pain symptoms
C	Urban, inland practice serving approx. 10,000 patients	Practice Improvement through increasing health literacy awareness Facilitating access to Financial Advice for patients Facilitating access to skills improvement (literacy) for patients
D	Rural, inland practice serving approx. 6,000 patients	Care Co-ordinator to work with patients to address barriers to accessing health care/prevention efficiently
E	Urban, coastal practice serving approx. 16,000 patients	Practice Improvement through increasing health literacy awareness Facilitating access to Financial Advice for patients Facilitating access to skills improvement (literacy, numeracy) for patients
F	Urban, inland practice serving approx. 8,000 patients	Cycling group to improve physical inactivity and increase access to green spaces, socialise and community cohesion Increase access to alternative mental health support (with local voluntary sector organisation) Welfare advisor at the Practice
G	Urban, coastal practice serving approx. 9,000 patients	Physical and Mental health worker to support patients with non-clinical needs
H	Rural, inland practice serving approx. 3,000 patients	A care co-ordinator dedicated to the practice to address needs of the patients
I	Rural, inland practice serving approx. 3,000 patients	A care co-ordinator dedicated to the practice to address needs of the practice
J	Urban, inland practice serving approx. 13,000 patients	Cycling group to improve physical inactivity and increase access to green spaces, socialise and community cohesion Increase access to alternative mental health support (with local voluntary sector organisation) Welfare advisor at the Practice

^*^Data on patient population reflects PCN adjusted populations for 1 January 2025, and data derived from Organisation Data Service's (ODS) ePCN publication https://digital.nhs.uk/services/organisation-data-service/data-search-and-export/csv-downloads/gp-and-gp-practice-related-data

Within these initiatives, our exploratory analysis identified three discernible trajectories through which practices’ situated the ‘opportunity’ presented by CareDEEP. The three trajectories were:

- efforts to enhance the uptake of preventative measures and improve engagement with healthcare through care navigation.
- strategies aimed at improving the accessibility of general practice services; and
- actions directed toward influencing the broader social and structural conditions affecting patients’ lives.

The four practices that engaged with prevention and care navigation appeared to focus exclusively on prevention and care navigation, with initiatives aimed at increasing screening uptake, immunisation, and care coordination. In contrast, the two practices that prioritised improving access to general practice services sought to impact on patients’ broader social contexts through initiatives focusing on skills development and welfare advice. The four practices whose initiatives solely targeted the social conditions of patients’ lives adopted more holistic approaches, ranging from forming peer support groups, to tailored, individualised interventions addressing multiple dimensions of social need. The process of adopting any given trajectory for intervention development by a specific practice appeared to be shaped by their understanding of social determinants of health, assessments of patient capacities, and evolving conceptions of general practice’s remit within contexts of socioeconomic disadvantage. The following sections present the contextual features that appeared to shape the adoption of these three trajectories.

### Prevention and health navigation

Four practices (A, D, H, I) focused on improving uptake of preventative interventions and health navigation. Preventative interventions focused on cervical screening, bowel cancer screening, abdominal aortic aneurysm (AAA) checks, and lung health assessments. Health navigation tended to focus on facilitating attendance at routine health care appointments. The focus on prevention and health navigation appeared to be primed by an understanding of how to engage with social determinants within the constraints of their remit and perceived sphere of influence. Practices viewed the barriers to preventative care as firmly within the domain of social determinants of health and to be non-clinical in origin. In their view, tackling these enabled the practice to impact the social determinants affecting the patients’ health and wellbeing. The need for this approach, which aimed to enhance patients’ agency for proactive action, appeared to be primed by interlinked contextual features, which included influence and pressure from other healthcare organisations, perceptions of patients and the identity of general practice.

#### A higher calling

Interviewees indicated that their attention to low screening uptake was often prompted by external communications, particularly from Primary Care Networks (PCNs), which coordinate local general practice, and the Integrated Care Board (ICB), which oversees health services across the social system, highlighting disparities in screening participation within their patient populations. These external drives to improve screening uptake served as a catalyst for reflection and action, positioning missed screening opportunities as a priority area for intervention.

However, while recognising the need to increase prevention uptake, participants noted a mismatch between the design of national screening programmes and the lived realities of Deep End populations. Screening initiatives are typically aimed at asymptomatic individuals, yet many patients in DE communities face immediate and pressing health concerns that take precedence. One general practitioner argued that the physiological ageing of their patients outpaces chronological ageing, rendering age-based screening thresholds misaligned with local need:

“By the time someone’s 30 years old in my population, they’re 14 age years older than their numerical age... So at 30 they are a 44-year-old, at 44 they are 58, and so that plays a big part. A lot of these screening programmes are predominantly designed for older patients, but by the time my patients are older, they’re very old” (General Practitioner at Practice A).

This interviewee moved on to illustrate the workings of the structural undercurrents that underpin the design of prevention measures, highlighting their inability to design better-suited interventions for patients such as those in the Deep End.

“it’s not within our gift because that is something that’s done by the government. … There’s various things we try to push. But unfortunately... the game, if you will, is rigged. It’s stacked against deprivation; it’s stacked for the affluent and the well—that’s how it works” (General Practitioner at Practice A).

#### Patients living chaotic lives

In addition, Deep End patients appeared as a key dynamic in the narratives of the practitioners, who described their patients as experiencing complex and intersecting forms of disadvantage that significantly hinder engagement with preventative care. Patients were frequently characterised as experiencing barriers attributed to low literacy levels, unstable living conditions, multiple comorbidities, substance misuse, mental health challenges, and age-related vulnerabilities. These factors were seen to contribute to a pattern of living “from crisis to crisis,” undermining the capacity for proactive health care engagement.

‘’we’ve got a high deprivation area, very low employment area, there are a lot of mental health issues. And, because those people are struggling with their mental health, when you do manage to get them an appointment, they’re not always the best people attending. And, then, we get calls from them when they’re in crisis and, you know, the whole idea of these interventions is to try and make sure they have the ability to cope, …. So, when they’re not attending the meetings that we’ve managed to get them into, we’re just going from crisis to crisis to crisis.’’ (Practice Manager at Practice F)

Interviewees reflected on the cyclical nature of socioeconomic disadvantage and ill health, noting that deteriorating health often leads to downward social mobility, while poor social conditions further exacerbate health outcomes.

“the more ill you are, the more you fall down the social and economic ladder, and the more unwell you are in the first place, the less likely you are to get out” (General Practitioner at Practice A).

#### All that the practice could do

Despite recognising the mismatch between national prevention strategies and the lived realities of their patient populations, practices considered prevention and health care navigation as an important component of health care and their mandated role. Within this framing, CareDEEP offered an opportunity to direct focus to improving the uptake of prevention interventions and health care navigation.

“Unfortunately it’s like a game of casino and the house always wins so you have to do what you have to do, which is where it gets difficult. So … that’s where this project comes in because there’s no mileage for me from a business perspective doing the screening. I don’t get judged by it, it’s not a target that’s come from me because it’s a target that goes to the people on high…. We don’t manage the screening programme …. So what I’m finding with this is trying to find an admin person to do what essentially is an unfunded piece of work because I can’t allocate any funding to it because we don’t get any funding coming in for it.” (General Practitioner at Practice A)

A sense of necessity, coupled with limited flexibility in how prevention is operationalised, framed their engagement with CareDEEP. Within this context, CareDEEP was perceived as a resource that enabled two strategic pathways for action: patient education and care coordination—each offering a means to navigate the constraints of standardised prevention while trying to accommodate local needs.

The education pathway involved developing alternative models of communication to patients to promote screening uptake, reframing messaging to highlight the preventative value of participation. As one GP explained, the aim was to “flip the narrative” from reactive to proactive care, encouraging patients to attend chronic disease reviews and screening appointments as part of a broader strategy to maintain wellness.

‘’ (the) message is trying to say, “Look, please go for your screening, please come for your chronic disease reviews, please come and do these things because we’re trying to keep you well,” not the other way, so we’re trying to flip the narrative from being reactive to proactive.” (General Practitioner at Practice A)

The care coordination pathway focused on enhancing follow-up mechanisms for patients who did not respond to standard recall systems. This included a deeper inquiry into non-response patterns and personalised outreach efforts to ensure patients were informed and supported in making decisions about their care. As one practice manager described, the goal was to “exhaust all options” to ensure patients had the necessary information to make informed choices.

Both pathways aimed to build patients’ capacity to self-manage and navigate health care systems. Practitioners emphasised the importance of equipping patients with the knowledge and confidence to act independently, framing this as a sustainable approach to reducing crisis-driven care:

“if we can help one person not go into crisis and have a pathway that they can, you know, then understand who to contact, potentially, in a better circumstance than us. … that one person having the capacity, ability, knowledge to be able to deal with that themselves. …Give a man food and he eats for a day, teach him to fish and he eats for a month. That kind of scenario, in my head.” (Practice Manager at Practice F)

For the practices, CareDEEP enabled engagement to be tailored towards patients who were missing out on health care, either through missing cancer screening or through failing to engage with health care appointments, offering additional support which aligned with local needs and practitioner capacities.

### Improving access to healthcare

Two practices (C and E) focused their CareDEEP initiatives on improving the accessibility of general practice services. These efforts were shaped by three interrelated contextual elements.

#### Seeing health systems as complex systems

The decision to prioritise access emerged from practitioners’ awareness of the barriers patients face when navigating healthcare systems, particularly relating to low health literacy and language difficulties. Practitioners who pursued this approach cited health literacy of the patient population as a central concern, and the focus appeared to be primed through expertise in health literacy. In recognition of low health literacy among the patient population, the practitioners sought to approach this challenge by improving practice-level awareness and responsiveness, which they proposed would help them to simplify processes and procedures at their practices, leading to services being made more accessible to patients.

“There are a whole lot of people who might be struggling to access health care... and a lot about what we’re doing and how we can make it easier to increase the accessibility.” (General Practitioner at Practice D)

#### Patients experiencing complexity and disadvantage

The approach, which sought to simplify health care system, appeared to be developed in response to perceptions of patients’ circumstances. These practitioners described their patient populations as facing a constellation of intersecting challenges that complicate access to healthcare. These included socioeconomic disadvantage, mental health conditions, histories of physical trauma, educational disadvantage, and—particularly in areas with large non-English-speaking communities—language barriers. It was noted that, many patients living in their localities, especially those identifying as white British, had low literacy and numeracy skills, and this was attributed to systemic failures within the education system. These challenges were seen to contribute to a broader pattern of disengagement from healthcare services, reinforcing the need for practices to adapt their systems to better accommodate patient needs.

Practitioners acknowledged the deeply embedded nature of these barriers and the limited capacity of general practice to address them directly. Rather than expecting rapid transformation, they emphasised the importance of incremental, yet sustained efforts to improve accessibility. Within this context, CareDEEP was perceived as a resource that enabled practices to begin addressing these challenges in a locally responsive manner. Through CareDEEP, practitioners sought to simplify practice systems and build patient capacity, aiming to make healthcare more navigable and inclusive.

“we know that the more classic social determinants of health are very, very, very difficult to change. You can build people’s skills and you can simplify the system. So, the hypothesis is that we’ll be able to shift people along that dial, both by building some skills and also making the service easier to use” (General Practitioner at Practice D).

#### The role of general practice in addressing health literacy

Practitioners conceptualised health literacy as a dynamic interaction between individual capacities and the complexity of healthcare systems. Rather than placing the burden on patients to improve their literacy, they advocated for simplifying practice systems and communication strategies to ensure accessibility regardless of patients’ literacy levels.

“You work within the general practice to make the services, and the information we provide, and the access... accessible, whatever people’s level of literacy and numeracy.” (General Practitioner at Practice E)

Within this framing, CareDEEP was perceived as a resource that enabled practices to respond more effectively to the challenges their patients faced. By fostering organisational responsiveness to health literacy, practices aimed to improve patient experience, reduce missed appointments, and enhance clinical outcomes.

“It’s around the system working better for patients, so patients feel happier. Hopefully, that delivers in terms of clinical outcomes, but also things like complaints, and people not turning up.” (General Practitioner at Practice D)

### Impact of patients’ social living conditions

Four practices (B, F, G and J) used CareDEEP to develop interventions focused solely on the wider living conditions shaping patients’ lives, while two practices (C and E) incorporated elements addressing non-health related aspects of patients’ lives. Practices that focused exclusively on wider conditions explicitly recognised that many health challenges are rooted in complex, entrenched circumstances beyond the reach of conventional clinical interventions. Their approach indicated a shift in focus—from within the healthcare system to the social context—and appeared to be shaped by three interrelated contextual features.

#### An approach primed by recognition of socially embedded health challenges

Practitioners described a longstanding awareness that patients’ health is deeply influenced by socioeconomic conditions and adverse life experiences. They observed that clinical encounters often failed to address the underlying causes of distress, and in some cases, exacerbated it by pursuing biomedical explanations for socially rooted problems. This recognition prompted a call for more holistic, cross-sector approaches that could offer meaningful support beyond traditional healthcare pathways, as explained by this practitioner:

“When you work in a practice like mine it doesn’t take much to realise that you’re looking after people whose healthcare challenges and needs are heavily influenced by their social circumstances, their socioeconomic circumstances and poverty.… what we see all the time is people who come in feeling desperately unwell, and we tend to make them more ill by going hunting for physical causes that we’re never going to find. … Could we get better at identifying people with lived experiences that are deeply affecting their psychological world and their physical world and all the rest of it, and actually give them connectivity, support, something better than a trip to the hospital to help them with these problems?” (General Practitioner at Practice B)

CareDEEP was seen as an opportunity to facilitate such approaches, enabling practices to collaborate with voluntary sector organisations to develop non-clinical interventions.

#### Need for hope in the face of disadvantage and complexity

Practitioners described their patients as living in contexts shaped by profound social isolation, cumulative disadvantage and entrenched hopelessness. They observed that many of these individuals lacked meaningful social support, encountered systemic barriers to opportunities to improve their life chances, and had endured adverse life experiences, influencing both their engagement with services and their broader outlook on life. There was a shared recognition among practitioners that such complexity demands integrated, holistic responses and that narrowly focused, single-issue interventions were seen as insufficient.

“There isn’t one [issue]—patients are complex. And often, I don’t think we know our patients’ stories well enough to understand that complexity. That’s what I meant about the crossover. Use of medication… I know it’s a financially-based deprivation score that’s used. But I think, often, maybe with that, comes deprivation of opportunity, deprivation of outlook, and deprivation of support. And… childhood life circumstances, all ends up in this one person.” (General Practitioner at Practice J)

Practitioners also shared that effective interventions must foster hope and tend to both the emotional and practical dimensions of patients’ lives. Rather than approaching individuals through a deficit lens, some of them promoted an approach aiming to engage with the full complexity of patients’ lived experiences, offering support that was both compassionate and empowering.

“I see a lot of people, and they just go, ‘I don’t see the point of doing this. What’s the point? My life’s [], I’ve got no family, I’ve got no friends, I’m looking at these four walls every day.’ And if I can instil just the smallest bit of hope in them—to make them want to leave that flat, and come out, and do something… for me, hope is the biggest thing; that there is something better than what they’ve currently got.” (Social Prescribing Link Worker at Practice H)

#### General practice as a community asset

The practitioners who took this approach appeared to share a particular vision of general practice—one that foregrounds relationality, continuity, and embeddedness within the community. One described general practice as akin to a “corner shop” – a locally trusted institution characterised by familiarity, attentiveness, and informal surveillance of wellbeing, who elaborated:

“One of the issues in primary care, or general practice, is that we are corner shops, aren’t we? And actually, that is the model that is within general practice… there are lots of really important parts of corner shops, aren’t there… because it’s that relationship model… where the old person doesn’t go in, two days in a row, who then goes, ‘Oh, I haven’t seen Mrs Smith.’ And these are really important within society… that smaller corner shop, that trusted relationship.” (General Practitioner at Practice J)

This relational model of care was perceived to be under threat, with practitioners expressing concern about broader systemic pressures that risk eroding the foundational values of general practice, which one practitioner described as “there seems to be a big push to lose [this model] within primary care.”

Within this context, an opportunity to engage with social determinants of health was seen as an opportunity for reclaiming and reimagining general practice in ways that prioritised holistic, person-centred support. For some, this involved collaboration with Voluntary, Community and Social Enterprise sector partners to deliver goal-oriented support via a link worker, enabling patients to navigate complex challenges and build personal agency. For others, the initiatives took the forms of welfare advice, community activities to enhance social connection, skills development, and accessible service delivery that meet patients “where they are,” both physically and emotionally.

Practitioners anticipated that such approaches would enhance patients’ quality of life not only through clinical outcomes, but also through increased confidence, reduced isolation, and strengthened capacity to manage everyday challenges. By fostering flexible, personalised, and locally embedded support systems, these practices appeared to offer care that was responsive to the lived realities of individuals in socioeconomically disadvantaged communities.

**Fig. 1:**
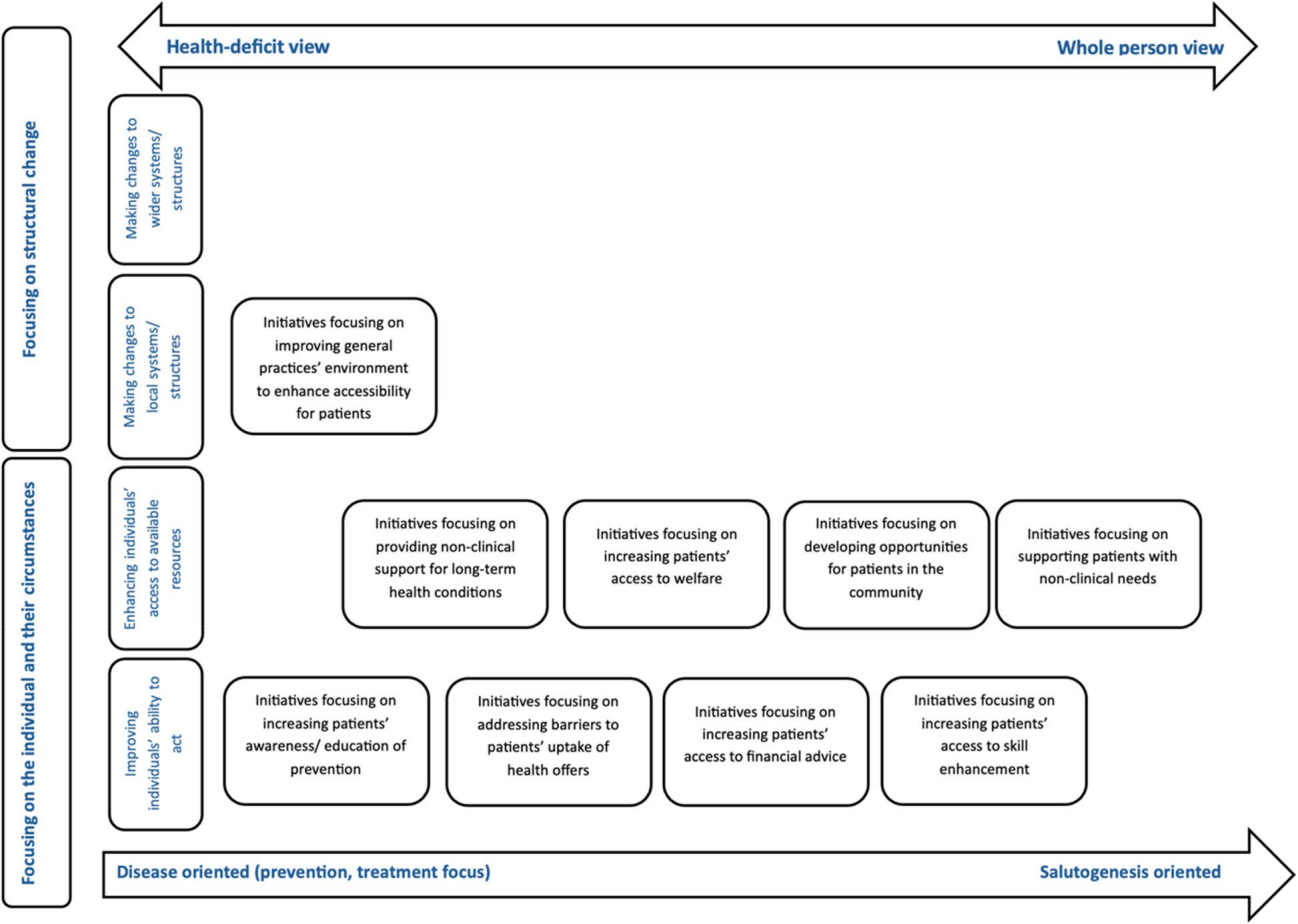
Pathways of impact of CareDEEP initiatives

Our findings indicate that the actions practices initiated in response to CareDEEP entered discourses on social determinants at diverse entry points. Figure 1 illustrates these actions, positioned across two intersecting dimensions: a continuum from a health-deficit, disease-oriented perspective to a whole-person, salutogenic perspective, and a gradient from interventions focused on individual circumstances to those targeting local and social structural change. The map situates the range of initiatives—including prevention and uptake of health offers, access to welfare and financial advice, non-clinical and community-based support, skills development, and changes to practice or system environments—illustrating how general practice activity could vary in scope, orientation, and potential to address health inequalities.

## Discussion

This study examined how general practices in socioeconomically disadvantaged areas responded to a locally driven initiative to address the social determinants of health. Consistent with international research, we found that practitioners drew on diverse discourses to make sense of health inequalities and their role in addressing them [1, 18]. However, by applying a contextual lens, our analysis extends this literature by showing how specific contextual configurations—comprising practitioner beliefs about the nature of disadvantage, perceptions of patient agency, understandings of general practice’s remit, and relationships with the wider health system—shaped the development of CareDEEP interventions.

Rather than viewing disadvantage as a static backdrop, we conceptualised context as a dynamic set of relations that shape what actions appear possible, legitimate, and meaningful for general practice [9, 13]. Through this lens, we identified three distinct logics of action that underpinned practices’ responses: a prevention-oriented logic focused on mandated priorities and patient activation; an organisational responsiveness logic centred on health literacy and system navigation; and a relational, community-embedded logic oriented toward the wider social conditions of patients’ lives. These logics illuminate how practitioners translated the broad call to address social determinants into situated forms of action as summarised below.

### Prevention and patient activation

Practices that prioritised prevention and care navigation enacted a logic grounded in a contextual configuration comprising of external performance pressures, national screening priorities, and perceived barriers to patient engagement, where social determinants were interpreted primarily as obstacles to the uptake of biomedical interventions. The resulting initiatives—enhanced recall systems, patient education, and care coordination—reflected mechanisms which the practitioners viewed as feasible to address the obstacles within existing constraints. This aligns with functional orientations in primary care, where it appears that institutional mandates and resource constraints narrow the scope of action to downstream, individual-level interventions.

### Organisational responsiveness

A second group of practices adopted a logic focused on improving access by simplifying organisational processes and enhancing health literacy responsiveness. Here, understandings of health care system and patients’ lives as complex and cumulative influenced a view of social determinants which highlighted a mismatch between patient capacities and demands of healthcare systems. This logic positioned general practice as an organisation that must adapt to patients’ capabilities to reduce barriers to care. The interventions developed—such as health literacy training and system redesign—reflect an analytical orientation that recognised how healthcare structures impacted on access and sought to modify organisational practices to improve equity.

### Relational and community-embedded practice

Practices that focused on patients’ wider living conditions enacted a relational logic that positioned general practice as a community asset embedded within local social worlds. Informed by a notion of disadvantage as cumulative and interconnected, this form of action on social determinants foregrounded hope, connection, and holistic support, in the context of entrenched, inescapable conditions. Interventions such as welfare advice, community activities, and voluntary-sector partnerships reflected an aspiration to address the social roots of ill health, even when the practitioners perceived that structural change required to address SDoH effectively remained beyond the immediate reach of general practice. 

### Diverse logics of action converging on individual-level responses: potential for tackling SDoH

Foregrounding logics of action helps explain why practices responded differently despite facing similar socioeconomic conditions. The three logics illustrate how varied contextual configurations shape not only what practices do, but how they understand their role in addressing health inequalities. However, while the logics and the ensuing pathways varies, as Fig. 1 illustrates, most interventions developed through CareDEEP converged on a singular focus on individual-level action. This aligns with previous research indicating that healthcare professionals’ accounts of SDoH are often marked by inconsistencies, with structural factors acknowledged but frequently subordinated to individual-level explanations [19]. Our findings elucidate how the diverse framings impact action on SDoH. For instance, approaches centred on patient activation used within prevention and health promotion initiatives may risk further disengagement among those facing the greatest barriers, as they do not address the structural determinants that shape patient experience and engagement [3, 17].

Our findings also challenge the assumption that working in disadvantaged areas necessarily fosters a structural understanding of disadvantage. Many practitioners appear to conceptualise disadvantage as an additional layer of complexity, arising from the accumulation of immediate life pressures—such as housing instability, benefit-related difficulties, and competing priorities—often framed in terms of deficits in time, resources, and skills, rather than in relation to underlying structural conditions. This interpretation of disadvantage appears to give rise to a “we know what patients need” rhetoric, which is in turn shaped by broader policy discourses that prioritise proximal, behaviour-focused approaches to addressing health inequalities [2, 35]. Such surface-level explanations and associated actions risk overlooking the underlying drivers of health inequalities, thereby limiting engagement with the social determinants of health (SDoH). Previous scholarship has shown that health inequalities are patterned by socioeconomic conditions inclusive of income, wealth, power, and access across social groups, and that access to key assets—such as material, biological, psychological, social, cultural, and spatial—shaping health and longevity is structured by class and command relations [21, 34]. Within these circumstances, a continued focus on downstream interventions, while overlooking upstream structural drivers, has been highlighted as having limited impact and described as a “fantasy paradigm” [35].

The diversity of logics, however, points to a transformative space through which general practice could play a key role in addressing SDoH. Despite the differences, practitioners presented a shared recognition that new approaches are needed for people experiencing socioeconomic disadvantage. GPs already demonstrated potential for action at the micro level of service delivery, particularly through recognising the need to tailor care and expressing a desire to position general practice as a community-oriented body that builds relationships and multi-agency partnerships to address barriers to health. This potential could be further strengthened through structural competency, enabling GPs to better understand how social determinants shape patients’ lives and to identify more effective points of engagement [24]. Such capacity-building would support GPs to take a more active role in mitigating the impacts of adverse determinants, while remaining attentive to what is feasible across patient, community, and policy levels [1, 15].

### Implications for practice and policy

Action-oriented learning initiatives such as CareDEEP may play an important role in strengthening practitioners’ structural competency by creating space for reflection, experimentation, and exploring new ways of working, These findings suggest that flexible, context-sensitive initiatives can help general practice to carve out a role where they could contribute more meaningfully to efforts to reduce health inequalities.

However, realising this potential requires structural and ideological support. Policies that prioritise relational care, community partnerships, and organisational flexibility may enable practices to move beyond reactive service delivery toward more sustained engagement with the social determinants of health. Supporting practitioners to navigate the generative space between clinical service and community anchor is essential for developing contextually grounded, equity-oriented models of care. Within the UK context, where policy is shifting towards neighbourhood health, it is critical to consider how context—encompassing resources and underlying ideologies—shapes action. In practice, this means that efforts to implement neighbourhood models should attend to resource allocation, professional assumptions about responsibility, patient engagement, and the role of general practice influence service design and delivery.

### Limitations

This study is subject to several limitations, both in relation to the CareDEEP intervention itself and the scope of the research. Firstly, the design of CareDEEP—while intentionally flexible and bottom-up—was constrained by limited funding and a short implementation period. These constraints may have shaped the nature of the initiatives developed, potentially limiting the depth and sustainability of practitioner engagement. In some cases, general practices reported that the scale of support restricted their capacity to pursue more ambitious or structurally oriented interventions.

Second, the study is affected by pilot bias. The practices that participated in CareDEEP were self-selecting and may represent a subset of practices that were already inclined toward innovation or community engagement. As such, the findings may not be generalisable to the broader population of Deep End practices, particularly those facing more acute resource constraints or differing ideological orientations.

Third, the research itself was bound by time and capacity limitations. While our approach enabled a nuanced exploration of how context shaped action, the loosely defined nature of the intervention meant that the analysis focused on emergent outcomes rather than predefined indicators of success. This exploratory orientation, while appropriate for the study’s aims, may have led to the underrepresentation of certain dimensions—such as the long-term relevance or effectiveness of specific pathways for local populations.

Finally, while the study foregrounds contextual variation, it does not offer a comparative analysis across different regions or health systems. Future research could build on these findings by examining how similar initiatives operate in other socio-political contexts and within different health systems regionally, nationally and internationally, and by exploring the conditions under which general practice can more fully engage with social determinants of health.

## Conclusion

The CareDEEP initiative provided a platform for DEN NENC practices to develop locally responsive interventions, revealing a spectrum of discursive orientations and strategic adaptations. While most interventions focused on individual-level support, they nonetheless reflected a commitment to addressing the social complexity underpinning health inequalities. This study demonstrates that general practice–led engagement with the social determinants of health is shaped not only by practitioner ideology, but also by the specific contextual configurations within which action is situated.

Importantly, the findings challenge assumptions that working in deprived areas automatically confers structural insight. Instead, disadvantage was often conceptualised as an additional layer of complexity rather than as a root cause. The equivocality in how practices interpreted their role—oscillating between service provider and community anchor—suggests a generative space for reimagining general practice. Within this space, initiatives such as CareDEEP can foster structural competency, relational care, and grassroots engagement, offering a pathway toward more equitable and contextually grounded healthcare.

Future efforts to support general practice in addressing health inequalities should prioritise flexible, context-sensitive approaches that recognise the diversity of practitioner perspectives and the relational nature of care. Structural and ideological support is essential to enable practices to move beyond reactive service delivery and towards sustained, systemic engagement with the social determinants of health.

### Reflexivity statement

This study was conducted by an interdisciplinary team comprising an anthropologist (lead author), general practitioners, public health specialists, and methodological researchers. The lead author is an anthropologist from an ethnic minority background who was not born in the UK. Her work is informed by a commitment to social justice and a sustained interest in how structural inequalities are reproduced or contested through everyday institutional practices. This orientation shaped the study’s focus on how general practices interpret and operationalise action on the social determinants of health (SDOH), and on examining the “logics of action” underpinning implementation rather than evaluating interventions solely in terms of outcomes.

Her positionality—both as someone trained to analyse systems and discourses, and as a researcher situated outside dominant UK social and professional hierarchies—sensitised the analysis to issues of power, institutional constraint, and the ideological framing of health inequities. At the same time, this orientation carried a risk of privileging structural explanations; this was actively examined through reflexive discussion within the team.

Co-authors who are practising GPs and public health specialists brought lived experience of working in socioeconomically disadvantaged settings, grounding interpretations in the realities of workforce pressures, contractual constraints, and local partnership infrastructures. Methodological colleagues supported analytic rigour and reflexive scrutiny of assumptions, particularly in applying realist-informed analysis and theoretical frameworks on SDOH discourses and approaches to action. Differences in disciplinary background and professional positioning were treated as analytic resources. Regular team discussions were used to interrogate interpretations, surface implicit assumptions about primary care’s role in addressing inequity, and ensure that findings reflected both structural critique and practical context.

## Supplementary Information

Supplementary Material 1

Supplementary Material 2

## Data Availability

The data generated and analysed during this study are not publicly available. Qualitative data, including interview transcripts and observational fieldnotes, contain information that could potentially identify participants despite anonymisation, given the small number of participating practices and the specificity of the research context. Participants consented to their data being used for the purposes of this study only. Ethical approval was granted for a defined study protocol, and public data sharing was not within the scope of that approval. Requests for further information about the data may be directed to the corresponding author.

## Abbreviations

SDOH: Social determinants of health
DEN NENC: Deep End Network North East North Cumbria
EOI: Expression of Interest
PCN: Primary Care Network
ICB: Integrated Care Board

## Footnotes

1 For more information, see International Deep End, https://www.gla.ac.uk/schools/healthwellbeing/research/generalpractice/deepend/international/ last accessed Mar 12, 2026

2 For more information, see https://deependnenc.org, last accessed on Mar 12, 2026
