# Supplementary material for: Between the clinic and the community: a qualitative study of logics of action on social determinants of health in general practices serving disadvantaged communities
Source: BMC Public Health. 2026 May 28;26:2222. doi: 10.1186/s12889-026-27790-7 (PMC13403808; doi:10.1186/s12889-026-27790-7)
Supplement: Supplementary file 1 — Supplementary Material 1 [file 12889_2026_27790_MOESM1_ESM.docx]

**CareDEEP Wave 1 – Topic Guide**

**1. Introduction and Context**

Purpose: Set the scene and understand participant background

- Can you briefly describe your role and your involvement with CareDEEP?
- What was your practice/organisation hoping to achieve by engaging with CareDEEP?

Prompts:

- Level of involvement
- Type of practice/population served
- Previous experience with similar initiatives

**2. Motivation Behind CareDEEP**

Purpose: Explore why CareDEEP was developed and taken up

- What motivated your practice to get involved?
- What inspired you to focus on the problem you chose to address?

Prompts:

- Sources of information about local needs

**3. Experience of Developing CareDEEP**

Purpose: Understand how the participants understand and respond to the offer of CareDEEP

3.1 Development of Interventions

How did you go about developing your CareDEEP initiative?

What are your thoughts on the flexibility to develop locally tailored initiatives?

Prompts:

- Developing relationships within and outside practice
- Bespoke/localised approaches
- Autonomy in decision-making
- Understanding of patient needs

3.2 Funding and Resources

How did the funding influence what you were able to do?

How did the timeframe of CareDEEP affect your ability to develop your initiative?

What was your experience of the peer support aspect of CareDEEP?

How useful was the wider DE (Deep End) network?

Prompts:

- Ability to try new ideas without risk
- Constraints of short-term funding
- Action learning
- Sharing challenges/solutions
- Feeling part of a network
- Differences depending on similarity with other practices

3.3 Programme structure

What did you think about the monitoring processes within CareDEEP?

Prompts:

- Opportunities to reflect
- Usefulness for tracking progress
- Links to the DE network

What was your experience of the research support offered?

Prompts:

- Co-production of initiatives
- Support with evaluation
- Relevance depending on whether ideas were already formed
- Time constraints for engaging with researchers

**4. Challenges and Facilitators**

Purpose: Understand internal and external factors that have not been mentioned, that may have impacted the approach participants took to developing CareDEEP

4.1 Internal (Within Practice)

What were the main challenges and facilitators within your practice when developing CareDEEP?

Prompts:

- Staff capacity
- Leadership
- Existing infrastructure

4.2 External Factors

Were there any external factors that influenced your ability to implement CareDEEP?

Prompts:

- Policy environment
- Community resources
- Partnerships

**5. Sustainability and Future Directions**

Purpose: Explore long-term impact and learning

What aspects of your CareDEEP initiative have continued, if any?

What would need to happen for these interventions to be sustained long-term?

Prompts:

- Funding
- Workforce
- Integration into routine care

**6. Reflections and Recommendations**

Purpose: Capture learning and improvements

Looking back, what worked particularly well?

What recommendations would you make for future programmes like CareDEEP?

**7. Closing**

Is there anything else you’d like to add about your experience with CareDEEP?

*Notes for Interviewer*

Use open-ended questioning and follow participant language

Adapt order depending on flow

Probe for examples and specific experiences

Be attentive to differences between practices with/without pre-existing ideas
