# Supplementary material for: Between the clinic and the community: a qualitative study of logics of action on social determinants of health in general practices serving disadvantaged communities
Source: BMC Public Health. 2026 May 28;26:2222. doi: 10.1186/s12889-026-27790-7 (PMC13403808; doi:10.1186/s12889-026-27790-7)
Supplement: Supplementary file 2 — Supplementary Material 2 [file 12889_2026_27790_MOESM2_ESM.docx]

**Focus Group Topic Guide: CareDEEP**

*1. Experiences of CareDEEP*

Aim: Surface key contexts and variation across participants

Can you briefly describe what your practice did as part of CareDEEP?

Prompts:

- What kind of idea/intervention did you implement?
- Did you start with a clear plan, or develop it over time?

*2. Bespoke Approaches (Flexibility and Local Design)*

Initial programme theory to test:
Flexibility enables practices to design locally relevant initiatives, particularly when they already have ideas.

- Core question

What was your experience of having the flexibility to design your own initiative?

- Context probes

Did you already have a clear idea of what you wanted to do?

What was your capacity (time, staff, leadership) to develop something new?

- Mechanism probes

Did flexibility feel enabling (e.g., autonomy, creativity)?

Or did it feel challenging (e.g., unclear direction, pressure)?

- Outcome probes

What difference did this make to what you implemented?

Who did this approach work best for—and who did it work less well for?

- Group comparison prompt

Do others feel this worked better for some practices than others? Why?

*3. Funding and Sustainability*

Initial programme theory to test:
Funding reduces risk and enables innovation (e.g., hiring link workers), but short-term funding constrains meaningful implementation and sustainability.

Core question

How did the funding influence what you were able to do?

- Context probes

What were your staffing/resources like before the funding?

Were you able to recruit (e.g., a social prescribing link worker)?

- Mechanism probes

Did funding make you feel able to try new things without risk?

Did the short-term nature affect how you approached the work?

- Outcome probes

What were you able to achieve because of the funding?

What was difficult to achieve within the timeframe?

- Sustainability prompt

What has happened since the funding ended (or is ending)?

- Group comparison prompt

Does short-term funding affect practices differently depending on their starting point?

*4. Peer Support and Regular Communication*

Initial programme theory to test:
Peer support provides a space for shared learning and problem-solving, but is more effective when practices perceive commonality.

Core question

What was your experience of the peer support and regular communication?

- Context probes

Were other practices working on similar things?

How often were you able to engage?

- Mechanism probes

Did these spaces help generate ideas or solve problems?

Did they help you feel part of a network?

- Outcome probes

What changed (if anything) as a result of peer interaction?

When was it less useful?

- Group comparison prompt

What made peer support more or less useful across different practices?

*5. Monitoring and Reflection*

Initial programme theory to test:
Monitoring creates opportunities for reflection and connection to the wider network.

Core question

What did you think about the monitoring aspects of CareDEEP?

- Mechanism probes

Did it help you reflect on progress or adjust your approach?

Did it encourage engagement with the wider network?

- Outcome probes

Did monitoring influence what you did in practice? How?

- Group prompt

Did people use monitoring differently? What shaped that?

6. Research Support

Initial programme theory to test:
Research support enables co-production and evaluation, but is more useful for those without predefined ideas and when time allows engagement.

- Core question

What was your experience of the research support offered?

- Context probes

Did you already have a clear idea of your initiative?

Did you have time to engage with researchers?

- Mechanism probes

Did it help clarify ideas or build confidence?

Or did it feel less relevant to your needs?

- Outcome probes

Did it influence your intervention or evaluation?

Who was this most useful for?

- Group comparison prompt

Why do you think it worked better for some practices than others?

7. Cross-Cutting Reflections (Refining Programme Theory)

Aim: Explicitly test and refine CMO patterns

Thinking across everything we’ve discussed:

What were the key ingredients that made things work?

Prompts:

- Differences between practices with vs without initial ideas
- Differences in capacity, time, and staffing
- Role of programme components (funding, peer support, etc.)

8. Closing

If CareDEEP were to run again, what would you change to make it work better?
